# Determination of Local Electrical Properties Using a Potential Field Measurement for Electrically Conductive Carbon Fiber Reinforced Plastics with Metal Contact Pins Joined via Injection Molding

**DOI:** 10.3390/polym14142805

**Published:** 2022-07-09

**Authors:** Elisabeth Eckel, Klara Wiegel, André Schlink, Mohamed Ayeb, Ludwig Brabetz, Michael Hartung, Hans-Peter Heim

**Affiliations:** 1 Electrical and Electronic Automotive Systems, University of Kassel, 34121 Kassel, Germany; elisabeth.eckel@uni-kassel.de (E.E.); wiegel@uni-kassel.de (K.W.); ayeb@uni-kassel.de (M.A.); brabetz@uni-kassel.de (L.B.); 2 Institute of Material Engineering-Polymer Engineering, University of Kassel, 34125 Kassel, Germany; hartung@uni-kassel.de (M.H.); heim@uni-kassel.de (H.-P.H.)

**Keywords:** electrical testing, potential field measurement, electrical contact interface, X-ray microtomography, carbon fiber, polyamide 6, polycarbonate, assembly injection molding, anisotropic properties

## Abstract

Carbon fiber reinforced plastics (CFRP) bear a high potential in terms of electrical conductivity and its potential applications. A locally resolved electrical measurement method for these anisotropic materials is a key prerequisite for understanding the structural and manufacturing process-related interrelationships. The aim of this paper is to develop a measurement method that allows this to be achieved and also to investigate areas of overmolded metal contact pins in detail. CFRP samples with polyamide 6 and polycarbonate matrices were used, which were produced by using a custom-designed injection mold. In order to evaluate the measurement results and to correlate them to process related structural properties, reflected light microscopy and X-ray microtomography were used. Typical areas with significant fiber structures of assembly injection molded samples were electrically and structurally characterized to identify correlations. Among further results, the correlation of equipotential lines, acquired from the electrical analysis, with specific fiber orientations within the injection molded samples was demonstrated, fiber-poor areas were identified, and a beneficial influence of weld lines on contact resistances was determined.

## 1. Introduction

Carbon fiber reinforced plastics (CFRP) offer a high potential with regard to their applicability in many sectors, such as the automotive industry, aviation, as well as the sports sector. Weight reduction, high strength, fatigue properties and the possibility of component integration are just some of the many advantages [1,2]. Not only should the benefits of the mechanical properties be emphasized, but also the possibilities in the area of electrical applications. Due to the comparatively high electrical conductivity in the segment of conductive plastics, CFRP are well suited for electromagnetic shielding applications [3].

All polymer compounds modified to be electrically conductive by carbon-based fillers show anisotropic electrical properties. Their conductivity mechanisms, which are based on the formation of a conductive network, the percolation network, have been extensively investigated [4,5,6,7,8,9]. The formation of this percolation network is a fundamental requirement for electrical conduction through these anisotropic materials [10,11].

The electrical characteristics of such compounds depend largely on their filler properties, mainly on the specific conductivity, shape, orientation and concentration of the filler particles [12,13]. Some of these parameters are influenced by the injection molding process of fiber reinforced parts. In particular, the local orientations and filler concentrations should be mentioned here [14,15], as well as the potential formation of weld lines, which have an influence on the local fiber distribution [16,17] and thus also on the conductivity in this area. Due to processing dynamics, electrical properties may vary at different positions, which are placed in different flow situations in an injection-molded specimen [18]. There is also a correlation between the above-mentioned injection molding parameters and the electrical properties [19,20,21].

While the total resistance of such compounds is already well-studied [22,23,24], there are only a few approaches known that deal with the relationship of the local conductivity within such parts in connection with the filler orientation either by means of simulative or experimental methods [13,18,19]. In addition to determining the electrical properties of anisotropically filled polymer materials, the contact between such materials and metal parts is also a relevant topic. Approaches for the measurement of the electrical resistance of areas close to the contact as well as for modeling the contact mechanisms are available [18,25]. Direct overmolding of electrical metal contact pins by means of assembly injection molding offers particularly high potential for application scenarios involving the functionalization of injection-molded components.

Alongside conventional imaging methods for the structural analysis of fiber-reinforced polymer compounds, such as reflected-light microscopy, the non-destructive method of X-ray microcomputer tomography offers further benefits, especially for the determination of fiber properties. Hence, the analysis is not limited to a single cross-sectional plane, but can also include the evaluation of specific volumes. The polymer matrix and fibers can be segmented and the volumetric distribution of individual fibers of different types can be quantified in three dimensions [26,27,28,29,30].

This paper provides a first step towards understanding the correlation of electrical contact resistances of overmolded contact pins and their local structural properties regarding the processing parameters. A new non-destructive method was developed to correlate electrical properties (like potential distribution) with filler properties (like orientation and particle distribution), and therefore with processing parameters (like mold temperature or injection pressure). This is done by means of a surface potential measurement.

The innovation of this method is the possibility of determining local electrical properties within anisotropically conductive specimens, such as CFRP. This allows these local values and location-dependent characteristics to be directly linked to structural properties. Other standard methods can only determine volume or surface properties. This means that the properties of the conductive network within the CFRPs are not taken into account.

The investigated specimens made of conductive CFRP (polycarbonate and polyamide 6) and an overmolded contact pin (gold-plated steel) in the center were joined via assembly injection molding. This pin also acts as an obstacle during the injection molding process and increases the variations of filler concentration and orientation in the compound. To present the method two different carbon fiber concentrations were used: 20 w% in polycarbonate and 30 w% in polyamide. All these factors lead to areas that differ greatly regarding their electrical properties.

These areas were investigated electrically and with image-based methods of structural analysis (light microscopy, X-ray microcomputer tomography).

## 2. Materials and Methods

### 2.1. Materials

#### 2.1.1. Polymer Compounds

Two different plastic compound types were used for the sample preparation. The first one is an amorphous polycarbonate with 20 wt.% carbon fiber content (PC CF20; ALCOM PC 740/1.1 CF20, ALBIS, Hamburg, Germany). According to the supplier, this electrically conductive material for injection molding applications has a reduced surface resistance and high stiffness. The second electrically conductive modified compound used in this research consists of a polyamide 6 matrix and contains 30 wt.% carbon fibers (PA CF30; Durethan BCF30H2.0EF 900111, LANXESS, Cologne, Germany). The data sheet highlights the injection molding suitability and increased electrical conductivity. The parallel shrinkage of both materials is in a comparable range (about 0.15%), while the perpendicular shrinkage of PC CF20 lies between 0.1 and 0.3%, and that of PA CF30 is significantly higher at about 0.55%.

The following parameters of the filler particles of both materials were evaluated on injection-molded specimens. The diameter of the carbon fibers in both cases is approximately *d_f_* = 7.5 µm, as determined from scanning electron micrographs of a cryo-fractured samples. The fiber length was determined by dynamic image analysis (QICPIC/R06, Sympatec, Clausthal-Zellerfeld, Germany) and is approximately *l_f-PC_* = 240 µm for PC CF20 and *l_f-PA6_* = 185 µm for PA CF30.

The main differences between the compounds used are the carbon fiber content and length, the shrinkage behavior (perpendicular) and the structure of the polymer chain molecules (amorphous or semi-crystalline). By choosing these different filler contents (20 wt.%; 30 wt.%) and the different molecular structures (amorphous, semi-crystalline), the intended test method could be evaluated with a variation of thermoplastics from the initial stage without having to implement an extensive experimental plan in the development phase of the method.

#### 2.1.2. Contact Pins

**Figure 1:**
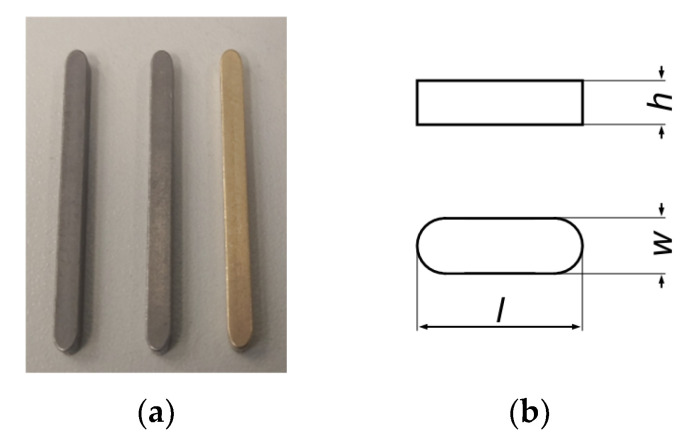
(**a**) Original, cleaned and gold-plated condition of the contact pins; (**b**) technical drawing of feather keys according to DIN 6885.

For the assembly injection molding of the test specimens, standard parts were used as electrical contact pins. These are feather keys according to DIN 6885 A with a square cross-sectional area made of tempered steel (C45K). A photograph of representative feather keys and a technical drawing can be seen in Figure 1.

The inserts must be as dimensionally accurate as possible to seal the cavity. For this reason, the dimensional accuracy of the present charge was examined on a random basis. The relevant data can be found in Table 1.

In order to prepare the surface of the contact pins for a sufficient electrical contact, they are coated with a gold layer using sputtering methods. A sputter coater (SCD 050, Oerlikon Balzers, Balzers, Liechtenstein) is used for this purpose. Before coating, all contacts are cleaned with acetone. The parameters of the coating process are shown in Table 2. To ensure uniform gold coating, the sputtering process is carried out on all four pin sides.

### 2.2. Sample Preparation

#### 2.2.1. Injection Molding

**Figure 2:**
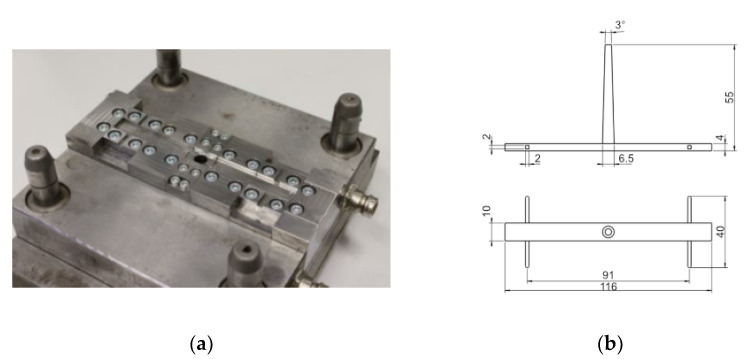
(**a**) Gate-side mold half of the modular injection mold. (**b**) Technical drawing of the specimen geometry.

The samples were produced by an assembly injection molding process. A special modular injection mold, which is shown in Figure 2a, was designed for this purpose. The inserts are held in position magnetically. This allows the integration of two contact pins, which are positioned to the gate equidistantly. Due to this positioning, both inserts are in the same flow situation during the injection process, resulting in equivalent structural properties. The resulting specimens can be viewed in Figure 2b.

Before processing, the polymer compounds PC CF20 and PA CF30 were dried in an air dryer (TR-Dry-Jet EASY 15, TORO-systems, Igensdorf, Germany) for approximately 4 h at 120 °C (PC CF20) and 90 °C (PA CF30). The specimens were manufactured with a hydraulic injection molding machine (Allrounder 320C, Arburg, Golden Edition, Loßburg, Germany). This injection molding machine utilizes a screw diameter of 25 mm and clamping force of 500 kN. The general processing parameters are given in Table 3. An open nozzle with an outlet diameter of 2 mm was used, since a valve gate nozzle in combination with the high processing temperatures can lead to excessive temperature and thus material degradation during the process. Preliminary tests have shown that this problem does not occur with the open nozzle. The packing pressure decreases linearly from packing pressure point 1 to point 2 over the given packing time.

#### 2.2.2. Specimen Preparation

**Figure 3:**
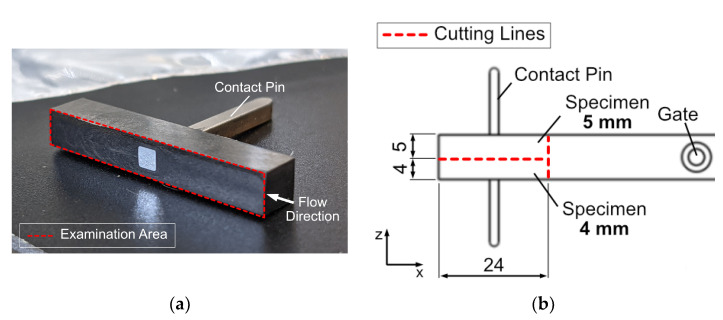
(**a**) Examination area on a prepared specimen. (**b**) Dimensions of a prepared specimen and cutting lines for precision sectioning.

For the subsequent optical imaging and electrical measurement methods, the examination area had to be exposed and prepared to obtain the specimens as shown in Figure 3a This requires cutting the specimens along the cutting lines shown in Figure 3b without disturbing the contact between the polymer compound and contact pin. A precision sectioning saw (IsoMet 1000, Buehler, Leinfelden-Echterdingen, Germany), which is particularly suitable for delicate specimens, is used for this purpose. The cutting speed is quite low, since the feed force is generated only by gravitation. This aspect, together with water cooling, prevents heating of the cutting surfaces despite the saw rotation speed of 350 rpm. The examination area was subsequently polished and cleaned in an ultrasonic bath to remove possible contamination by conductive carbon or insulating polymer matrix particles. As a result, two samples were obtained, which had a depth of 4 mm and 5 mm. The 5 mm sample represents the area exactly in the center of the specimen and the 4 mm sample, thus 1 mm below the center of the specimen. The specimens are referred to as PC CF20_4 mm, PC CF20_5 mm, PA CF30_4 mm and PA CF30_5 mm in the following.

### 2.3. Testing

#### 2.3.1. Reflected Light Microscopic Imaging

To obtain the reflected light micrographs, a digital microscope (VHX-600, KEYENCE, Osaka, Japan) with adapters for diffuse and coaxial illumination was used.

#### 2.3.2. X-ray Microtomography Analysis

For the analysis of the structural parameters of the anisotropic material, which are relevant for the electrical conductivity such as the fiber orientation and distribution in the contact pin area, X-ray microtomographic examinations were carried out. Specimens were prepared by removing the contact pin to enable higher contrast between polymer and carbon fiber phases. For the examinations, an X-ray microscope (Zeiss Xradia Versa 520, Carl Zeiss, Oberkochen, Germany) was utilized. A power of 5 W at a voltage of 60 kV was applied for the examinations. A total number of 1601 images were acquired with an exposure time of 6 s for each image and a binning setting of 1. The low energy filter LE2 and a magnification of 4× were selected, resulting in a voxel size of approximately 2 μm. Reconstruction of the individual images was performed within Zeiss XMReconstructor software. In addition, a 3D data visualization and analysis software system and the XFiber extension (Avizo 9.4, Thermo Fisher Scientific, Waltham, MA, USA) was used for further processing and evaluation of the three-dimensional data. Initially, the resulting volumetric data were aligned and filtered using non-local means filter before partial volumes were extracted at the areas of interest.

Cylinder correlation was used to characterize the fiber orientation in the specimens. To segment the fibers, a tracing algorithm was applied to the resulting correlation lines. The detected fibers can be quantitatively evaluated with regard to their fiber length and orientation in the test specimen. In the next step, the fiber tracing algorithm, provided by XFiber extension was applied to the subvolumes. Its settings are listed in Table 4. The values were adjusted to match the real fiber lengths determined by dynamic image analysis and the optical cross-sectional images from the µCT.

**Figure 4:**
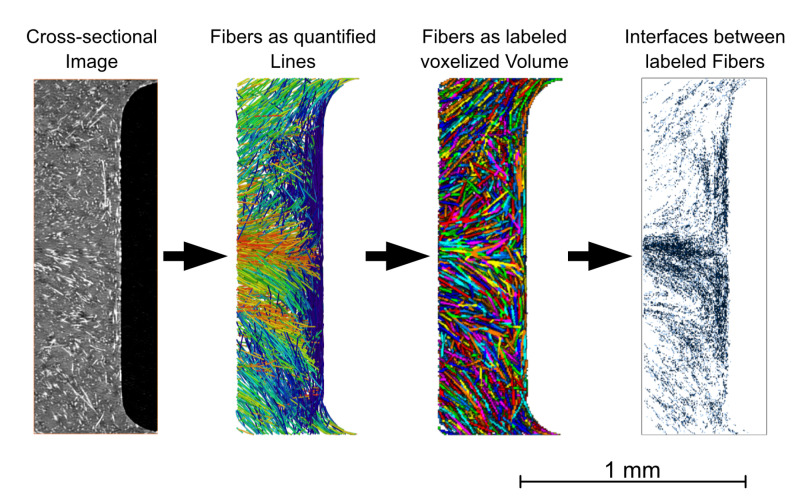
Evaluation method of the X-ray microtomography data.

This allows individual fibers to be quantitatively evaluated and displayed. The orientations of the fibers are specified by tensor values. Furthermore, the fibers can be labeled, and thus the contact points between the fibers can be visualized. An exemplary analysis procedure is shown in Figure 4, based on the weld line next to the metal contact pin. For the later examination, such results are taken from a xy-plane located at the center in z-direction with a layer thickness of 100 μm.

#### 2.3.3. Potential Field Measurement

As a first indicator for the electrical anisotropy of the samples, the total and partial resistances (*R_total_*, *R*_1_ and *R*_2_) of the specimen were determined, where *R*_1_ represents the weld line area of the sample and *R*_2_ the near gate area. Due to the positioning of the pin with regard to the melt flow progression during the injection molding process, different fiber orientations are expected in these areas and therefore different resistances, although the injected material is the same.

**Figure 5:**
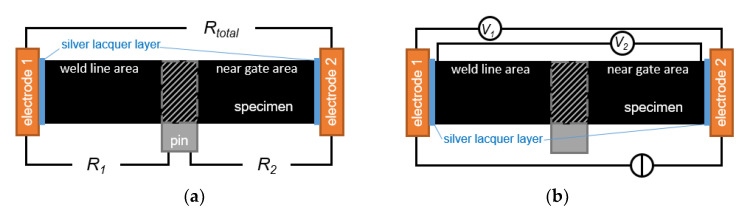
Schematics of the measurement setups in side view: (**a**) of the resistance measurements and (**b**) of the silver lacquer layer evaluation.

The most common method to measure potential differences and thus determine electrical resistances is the four-wire method. Figure 5a shows the measuring positions of theses resistances. To prevent a resistance change due to heating a low direct current of 20 mA was applied using a current source (PS 2016-050, EA, Viersen, Germany). The voltage measurement was done with a multimeter (34401A, Agilent, Santa Clara, CA, USA). Solid copper blocks (4 × 1 × 1 cm) were used as electrodes, which were designed and manufactured for this specific purpose. Due to the comparably low specific resistivity of copper and steel, the influences of the electrodes and the steel pin are neglected.

To ensure a good bonding of the conductive carbon fiber network to the electrodes, the terminal end surfaces of each specimen were coated with a thin layer of conductive silver lacquer (62900341 Ag Paste L204N, FERRO, Frankfurt, Germany), pressed onto the cleaned and ground electrodes, and dried for 1 h at room temperature.

To estimate the influence of this layer two potential differences were measured, see Figure 5b. *V*_1_ is the potential difference between the two electrodes, taking into account the specimen and the lacquer layer. *V*_2_ represents only the specimen and was measured close to the lacquer layer on the sample surface using contacting needles (S1E2D1, L-TRIS, Krailling, Germany). Although this method does not take into account a possible volume effect, since it is a superficial measurement, it can be used as a first approximation, if the potential difference and, thus, the influence of the volume are small. A difference between *V*_1_ and *V*_2_ could then be attributed to the lacquer layer. This measurement shows a negligible difference between *V*_1_ and *V*_2_, *V*_2_ is 0.2% smaller. Thus, the contact resistance between the specimen and the electrode can be neglected.

While the partial resistances *R_1_* and *R_2_* are a first indicator of the anisotropic electrical behavior of the samples, no further information regarding the local fiber distribution can be derived. However, the potential field measurement introduced below allows for a spatially resolved statement about the influence of this distribution based on a non-destructive electrical characterization of the specimens.

**Figure 6:**
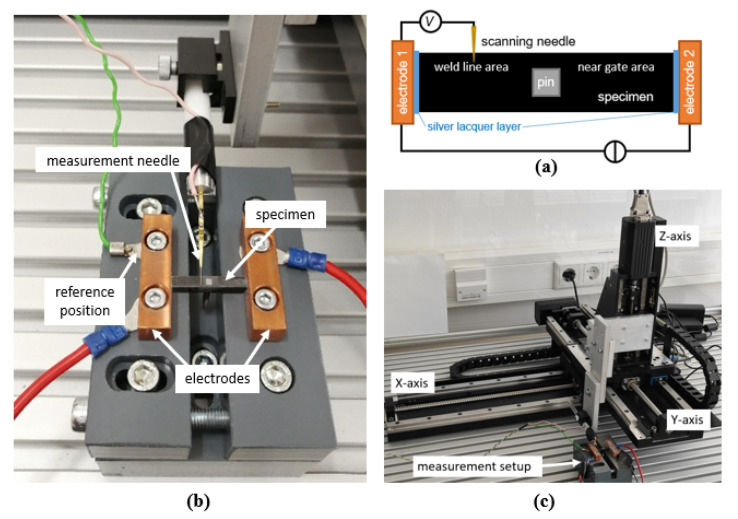
Potential field measurement setup: (**a**) schematic, top view; (**b**) implemented setup; (**c**) whole setup including the combination of cross and linear table used for positioning the scanning needle.

The potential field measurement is also based on the four-wire method. The measurement setup is shown in Figure 6. The used measurement devices are the same as before. A measurement needle with a tip diameter of ~13 µm (S1E1D1, L-TRIS, Krailling, Germany) scans the sample surface on a predefined area. The scanning of the surface is achieved using a cross table (*X*/*Y*-axis, KS15-450-390, ITK, Lahnau, Germany) and an additional linear table (*Z*-axis, ST9 eco, ITK, Lahnau, Germany). The positioning accuracies in the respective directions are shown in Table 5.

In general, it is expected that information about the conductivity anisotropy of CFRP can be obtained from the measurement results. For example, areas with a high potential gradient may indicate low conductivity. Significant potential steps even indicate high local resistances in the specimen.

## 3. Results

### 3.1. Flow Situation at the Examination Area

**Figure 7:**
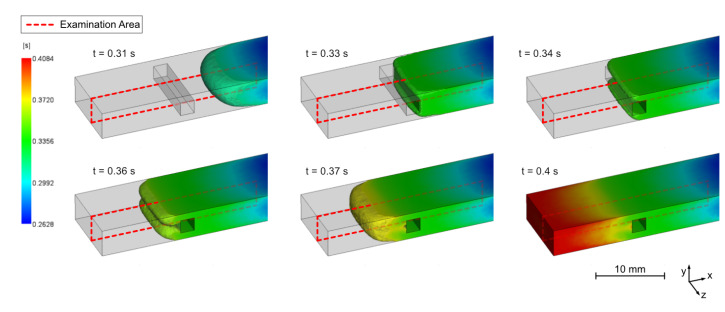
Melt flow obtained from injection molding simulation in the pin area showing the division of the melt front and the formation of the weld line after passing the pin.

To visualize the flow situation in the examination area, a basic injection molding simulation was carried out, using Moldflow Insight 2021 (Autodesk, San Rafael, CA, USA). For the simulation, precisely the sample geometry was used as shown in Figure 2b. The entire injection mold was not reproduced and implemented due to the illustrative study here. The materials PC CF 20 and PA CF30 were already defined with all properties in the Moldflow database. The recommended standard process parameters for these materials were selected and an analysis sequence (fill, pack, warp) was performed. The melt flow progression during the injection molding process of a PC CF20 sample is shown in Figure 7. In the illustration, the gate is located outside the image section on the right. Originating from there, the plastic melt flows through the rectangular cross-section and is not particularly influenced by the geometry of the cavity. Here, a fiber orientation according to classical layer models is formed. However, when the melt frontally hits the pin in the examination area, specific characteristics start to develop. By a continuous flow onto the pin surface until the entire volume is filled, the microstructure of the plastic compound is significantly influenced at this location. After the melt has hit the pin, it splits into two independent melt fronts as it passes through the side areas. At these side areas, the velocity and also the shear rate increase due to the locally reduced cross-section, which further determines the fiber orientations. Subsequently, they converge together again and flow as a combined front up to the end of the flow path. This results in a typical flowing weld line, which in turn leads to characteristic properties with regard to the microstructure.

**Figure 8:**
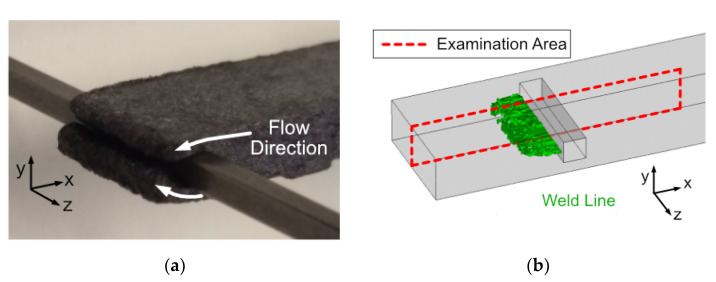
(**a**) Melt front dividing irregularly at the pin area of an injection molded sample (PC CF20); (**b**) representation of the emerging weld line at the pin area obtained from injection molding simulation.

The simulation result shows a uniform merging of the flow fronts after passing the pin, as also shown by the uniform weld line shown in Figure 8b. However, this uniform shape can only be expected to a limited extent in a real injection molding process with highly filled CFRP.

A sample from a flow study, shown in Figure 8a, proves that the split flow fronts do not pass the pin sides at exactly the same speed. Those two flow fronts do not progress evenly passing the insert section. The gate position contributes to the problem, since the melt first impacts on the mold side opposites the gate, which can lead to an advanced filling of the volume and a higher pressure in the mold half on the closing side. At the same time, a slight difference in the tempering of the mold halves can also have an influence on the flow rates of the two narrow sections.

Due to the constriction and the high pressure present in the process here, a formation of a weld line can occur, which means that irregularities in the fiber orientation in the volume behind the insert can be expected. Free-jet formation also contributes to the development of the weld line occurrences. These irregularities cannot be avoided in this case, as filling of the cavity cannot be guaranteed at lower pressures.

**Figure 9:**
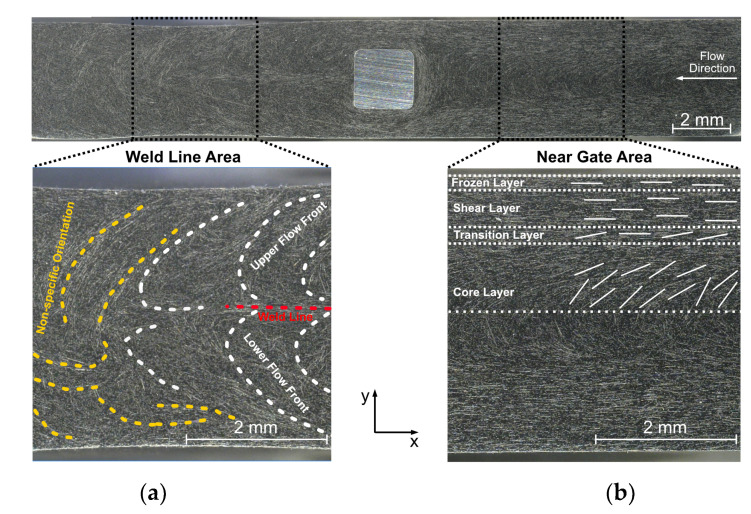
Light microscopic illustration of the examination area (PC CF20_4 mm specimen) with focus on near gate area (**b**) and weld line area (**a**) with schematically marked characteristic fiber orientations.

The reflected light microscope image in Figure 9 allows an analysis of the fiber orientations in the examination area caused by the injection molding process. Since the essential structures in the two examined planes (4 mm and 5 mm) of both materials (PC CF20 and PA CF30) show a similar formation, these phenomena, which are relevant for the further investigations, are explained based on the example of a PC CF20_4 mm specimen.

The schematic indications shown in Figure 9 can be used to identify characteristic fiber structures before and after the melt flow passed through the pin region. In the near gate area, orientations as known from layered models are recognizable. Typical fiber orientation regions resulting from the source flow can be identified here. Layers are established, which are oriented to known layer models [31,32]. The most important of these are the shear layer, which primarily contains fibers oriented in the flow direction and the core layer, where the fibers are aligned along the progressing flow front.

In the weld line area, the orientations are determined by the weld line formation and its implications. A straight aligned weld line can be identified in the immediate proximity behind the contact pin (red), which was to be expected as a goal of the cavity design. Weld lines cause locally increased fiber concentration and a strong orientation of the fibers in x-direction due to the encounter of the flow fronts [17,31].

In the more distant areas (non-specific oriented areas; yellow), there is no distinct weld line appearance, but the fronts also flow together, but less predictably. This is related to the formation of free jets. These do not develop like a source flow, but enter the free volume in an unguided manner and fold into each other, so that they subsequently also cause weld line-like structures, which are recognizable, but not reproducible.

### 3.2. Electrical Resistances

The resistance measurements provide some interesting results (Table 6). The resistances of the PC CF20_4 mm specimen are slightly higher than that of the 5 mm counterpart. This is to be expected since the cross-sectional area of the 4 mm specimen is smaller, resulting in a higher resistance when the same material is used.

**Figure 10:**
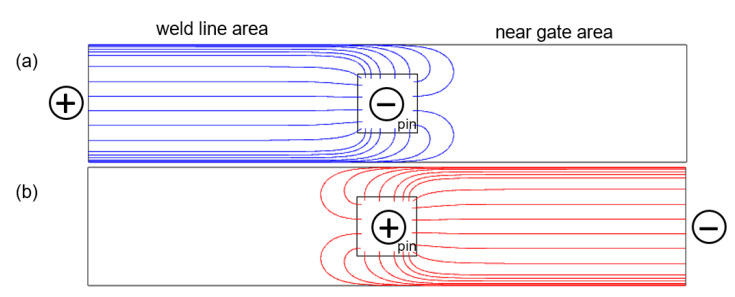
Simplified schematics of the current paths for the measurement of the partial resistances (**a**) *R*_1_ in blue, and (**b**) *R*_2_ in red.

The sum of *R*_1_ and *R*_2_ is about 30% higher than *R_total_.* In part, this can be explained by the current paths. Figure 10 shows a simplified representation of the current paths for each measurement, assuming an ideal contact between the pin and the surrounding polymer matrix. Due to the geometry of the specimen, the measurement of one partial resistance always partially includes the other one, as well. This effect is expected to be small.

So far, an ideal contact has been assumed, but of course, there is also a contact resistance. This seems to be the main reason for the high partial resistances between polymer matrix and metal pin. Further information will be presented in Section 3.3.4.

The most interesting part is the difference between *R*_1_ and *R*_2_ for the PC CF20 specimens. As expected, it seems that different fiber orientations lead to different electrical resistances. In this case, the resistance of the weld line area is much higher than the near gate area. This is probably due to the non-specific, almost chaotic fiber orientation in this area. A more ordered fiber orientation seems beneficial for lower resistivity.

It should be noted that *R*_1_ consists of the resistivity of the weld line area and the contact resistance to the pin; and *R*_2_ of the resistivity of the near gate area and also a contact resistance to the pin. However, the contact resistances here can differ due to the current density distribution and fiber orientation on the left and right side of the pin.

Comparing these results with the measurements of the PA CF30 specimen, two things are immediately obvious: firstly, the total resistance of the polyamide specimen is significantly lower (explained by the higher filler content) and secondly, *R*_1_ and *R*_2_ are almost equal; in fact, both are significantly higher than the total resistance. This is a clear indication of a poor contact resistance to the pin. A more detailed analysis of this circumstance follows in Section 3.3.4.

### 3.3. Potential Field Interpretation

**Figure 11:**
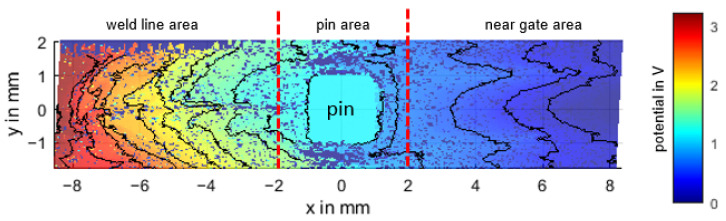
Potential field of the specimen PC CF20_4 mm with equipotential lines (potential difference between the lines is 0.2 V). The top left area is outside of the sample.

Based on the measurement of the specimen PC CF20_4 mm (Figure 11) the main characteristics of the obtained potential field will be discussed.

#### 3.3.1. Areas with No Connection to the Conductive Network (Non-Contact Areas)

If the measuring needle reaches an area that is not connected to the conductive carbon fiber network, the measuring circuit is not closed and 0 V is measured accordingly. This can occur in areas with a low filler concentration or unfavorable filler orientation, where the carbon fibers do not reach each other. Small areas with no contact are scattered throughout the potential field. A fiber concentration of 20 wt% is probably not enough to allow good crosslinking over the entire surface, leaving unconnected areas. Some of these areas are clustered along the weld line (probably due to air pockets, see Section 3.3.4) and larger non-contact areas are located below and above the contact pin (probably fiber depletion zones, see also Section 3.3.4).

#### 3.3.2. Near Gate Area

The potential difference of the near gate area is 0.8 V over 7 mm, which is about 25% of the total potential difference of the measurement field, indicating a better specific conductivity in this region. This agrees with the result of the resistance measurement. It can also be observed that the potential profile is smoother and more constant. This is probably due to the more ordered distribution of the fibers.

**Figure 12:**
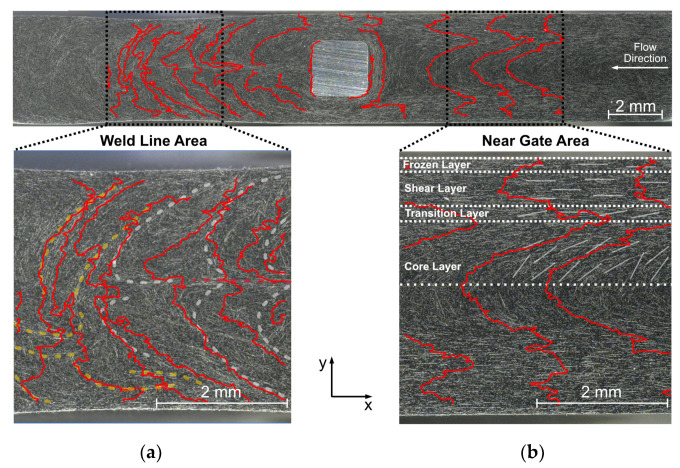
Transfer of the equipotential lines to the microscopy image with equipotential lines (red) and schematically marked characteristic fiber orientations (yellow, white) with focus on near gate area (**b**) and weld line area (**a**).

The charge transport within such samples takes place via carbon fibers, as these have a much higher conductivity compared to the polycarbonate matrix. By definition, an equipotential line is perpendicular to the current density flow, so one would expect the equipotential lines to be perpendicular to the fiber orientation, as well. This mainly applies to the frozen and shear layer, because here the fiber orientation is aligned with the main current path (from electrode 1 to electrode 2, see Figure 12b). The conductivity depends on the specific conductivity of the carbon fibers and the contact resistance between connecting fibers. It should be noted that carbon fibers have two different specific conductivities, longitudinal about 10^4^ times better then transverse [32]. Since the contact resistance between two fibers can never be less than the resistance of the carbon fibers, a long current path along the fibers with few transitions at the contact points is preferable.

**Figure 13:**
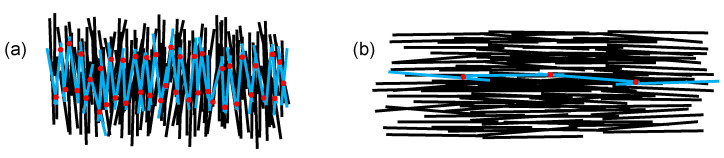
Two simplified scenarios for an electron path from left to right with a fiber orientation (**a**) transverse and (**b**) along the main current direction. The carbon fibers are marked in black, the selected electron path is highlighted in blue and the fiber contact points along the path are marked as red dots.

The transition and core layer, on the other hand, show a different behavior. Here, the equipotential lines follow the fiber orientation, indicating a current flow perpendicular to the fiber orientation, although the conductivity along the fibers is much higher. Current flow along the fibers is causing only a small potential drop due to the comparably high conductivity of the carbon fibers, the main potential drop is caused by the transition across the fibers in transverse direction. In addition to the already poor transverse resistance, the current path is also interrupted by much more transitions compared to a fiber orientation along the main current direction (Figure 13).

**Figure 14:**
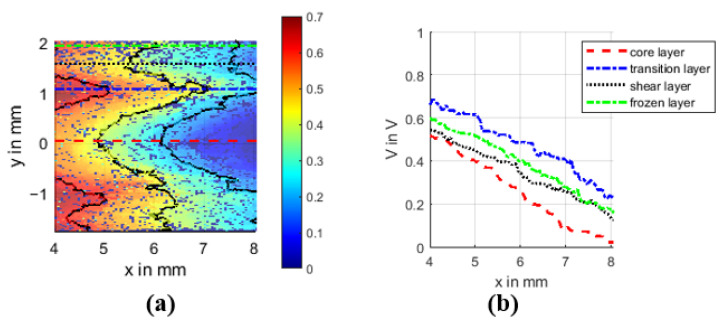
Potential field section of the near gate area (**a**), with marked positions of the potential profiles in (**b**) along these lines.

The distinguishable layers of the near gate area with regard to their potential field as well as the potential profiles along the center of each layer are shown in Figure 14.

All four potential profiles show a more or less linear potential drop with a comparable slope. According to Ohm’s law:

(1) J(x,y)=σ(x,y)E(x,y),

the current density ***J*** depends on the specific conductivity ***σ*** and the electric field strength ***E***. The material properties are strongly anisotropic due to the carbon fibers used. To represent this behavior, sigma must therefore be a matrix and thus location-dependent.

The comparable slope of each curve indicates a comparable ***E*** for each layer, and since the fiber orientation predicts a different ***σ***, the current density ***J*** must also differ. For the frozen and shear layer with a fiber orientation along the main current path and therefore good specific conductivity, the current density must be high for ***E*** to be equal. Conversely, the current density of the core and transition layer with a transverse orientation must be low. In fact, this is even worse for the core layer, since here the slope is slightly higher. Thus, as expected, the fiber orientation also affects the current density distribution.

To fully solve this equation, either ***σ***(*x*,*y*) or ***J***(*x*,*y*) must be known in addition to ***E***(*x*,*y*). This will be a topic for future studies.

#### 3.3.3. Weld Line Area

The weld line area has a potential drop of 2 V over 7 mm, which is about 63% of the total potential difference of the sample, compared to the near gate area the specific conductivity is worse here. The whole potential profile in this area seems to be rather chaotic compared to the near gate area, but a closer look reveals a strong dependence on fiber orientation. The equipotential lines seem to follow the fiber orientation (see Figure 12a), indicating a current flow perpendicular to the fiber orientation and therefore having many transition points, resulting in a lower conductivity of this area, consistent with the resistivity measurement (see Section 3.2). Since this area is rather chaotic and not reproducible, further analysis is omitted.

#### 3.3.4. Contact Pin Area

**Figure 15:**
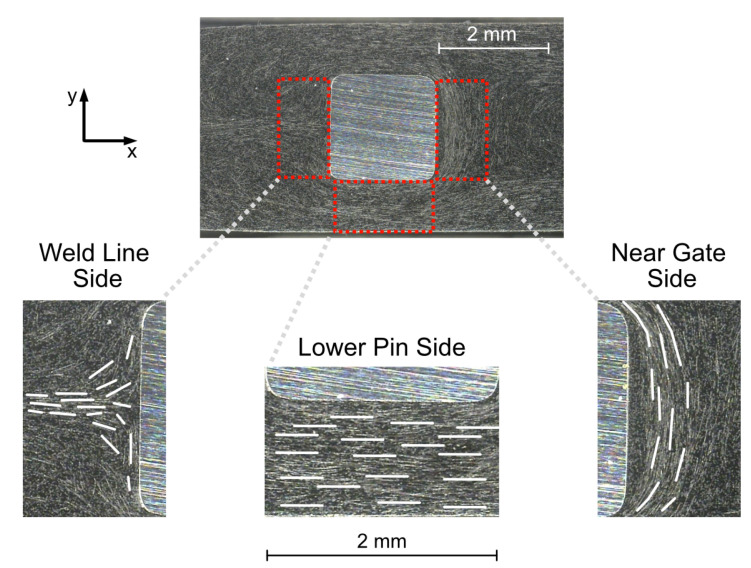
Pin sides according to a PC CF20_4 mm sample including indications for significant orientations.

For the analysis of the contact pin area, a distinction is made between three subareas, which differ primarily in terms of their fiber orientation in relation to the contact surface. Figure 15 provides the descriptions including their corresponding subareas.

Similar to the weld line area described in Section 3.1, the weld line side is also dominated by the weld line in the mid-section, which leads to increased fiber concentrations and an orientation perpendicular to the contact surface (at least at the center of the pin surface).

The microstructure of the lower pin side is mainly characterized by fibers that have been aligned by increased shear. The shear layer occupies an enormous proportion here, so that the fibers in this area are almost completely oriented parallel to the pin surface. Furthermore, increased shear rate is to be expected due to the narrow area. This leads to shear-induced particle migration at this position [33,34]. The carbon fibers arrange themselves predominantly in areas of lower shear, which leads to a reduction of the local fiber concentration in this area.

The third side to be considered is the one near the gate. Here, the melt flow front meets the surface directly. Additionally, at this point, the separation of the fronts takes place. Until the volume behind the insert is completely filled, the melt flows continuously onto the surface. The orientation near the contact surface cannot be predicted clearly, but a low orientation is likely. The effect of shear-induced particle migration also plays a role here since the shear stress is increased. On top of this low-orientation layer, carbon fibers are increasingly attached, which form a parallel layer in relation to the pin surface.

**Figure 16:**
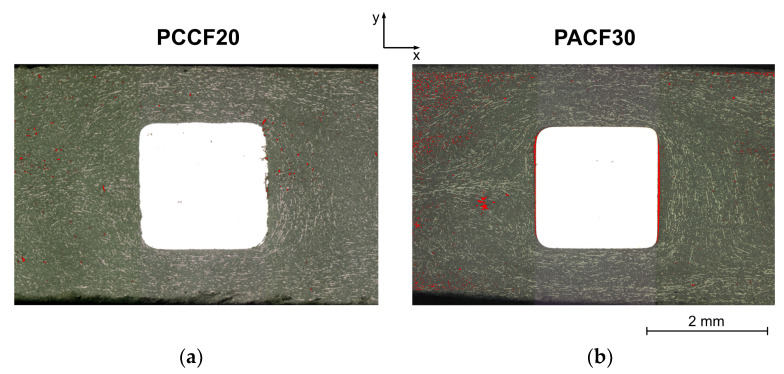
Microscopic images with marked air pockets and voids (red) of the pin areas of the specimen PC CF20_5 mm (**a**) and PA CF30_5 mm (**b**).

The microscopic image in Figure 16 was obtained in order to visualize irregularities of the prepared surface and thus to identify air pockets and voids.

Especially in the case of the PA CF30 specimen, voids can be detected around the weld line; for the PC CF20 specimen they are more scattered. Gap formation between the pin surface and the plastic compound can also be observed on the near gate side and the weld line side for PA CF30. In comparison, this observation is only present to a minor extent in the PC CF20 specimen. This can be explained by the volume shrinkage of the two materials. The perpendicular shrinkage of PA CF30 is almost twice as high as that of PC CF20.

**Figure 17:**
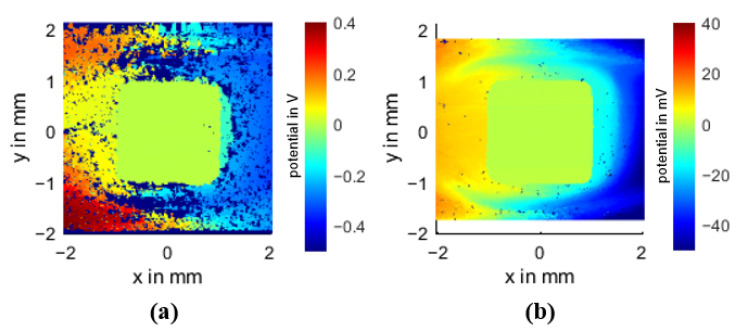
Measured potential fields of the pin areas of the specimen PC CF20_4 mm (**a**), and PA CF30_5 mm (**b**).

The described filler orientations of the three subareas of the contact pin area shown in Figure 15 are now correlated with the electric potential field (Figure 17). In order to characterize these areas more precisely, X-ray microtomographic examinations were evaluated and compared with the measured potential fields. In general, it is noticeable that there are significantly more non-contact areas (for the PC CF20 specimen). To explain why this is the case, and to identify other interesting features, the three subareas around the pin are considered individually and compared with the fiber orientation there.

**Figure 18:**
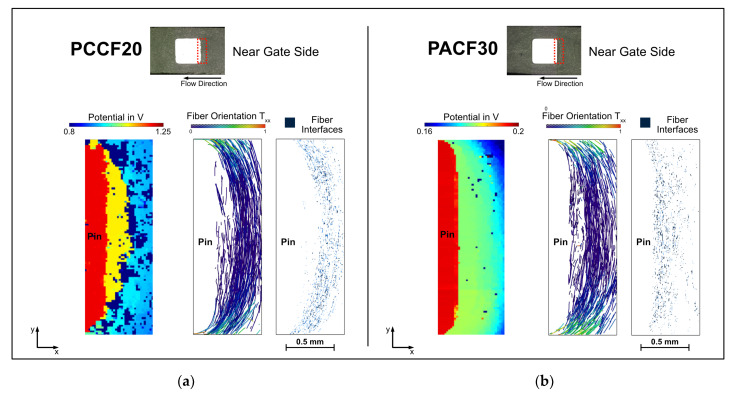
Comparison of the measured potential fields and the fiber orientation at the near gate sides of the specimen PC CF20_4 mm (**a**) and PA CF30_5 mm (**b**).

On the near gate side of the PC CF20 specimen, there is a clearly identifiable layer in front of the pin, exactly at the position of the prior identified low orientation layer (Figure 18a). This layer is at the same potential and is clearly separated from the pin (potential difference of ~160 mV) and the rest of the surrounding material by a potential step (potential difference of ~150 mV). This indicates a poor connection to the other areas. There is also a clear difference in fiber orientation between this layer and the rest. This layer shows a comparably low fiber concentration, but there are almost no non-contact points. Consequently, the carbon fibers within this layer, although not many, are well connected and present everywhere, at least at the measurement surface. The carbon fibers directly in front of the pin are all aligned parallel to the pin surface, probably the reason for the potential difference between pin and the low orientation layer, indicating a poor electrical bonding. The connection between this layer and the surrounding material is also poor, indicated again by a potential difference and the presence of many non-contact areas in between.

In order to investigate the influence of the filler content on the bonding to the metal pin in more detail, this result was compared to the measurement of the specimen PA CF30_5 mm (Figure 18b). In this measurement, the layer in front of the pin is not so clearly electrically separated, but clearly better bonded to the rest of the surrounding material. The bonding of the carbon fibers to the pin is again poor; the carbon fibers are again aligned parallel to the pin. In addition, shrinkage of the material occurs in front of the pin (Figure 16), and is probably the reason for the potential difference here. Based on these two results, it can be concluded that for this material combinations an electrical bonding at the near gate side seems to be not optimal, although the potential difference for the higher filled PA CF30 is.

**Figure 19:**
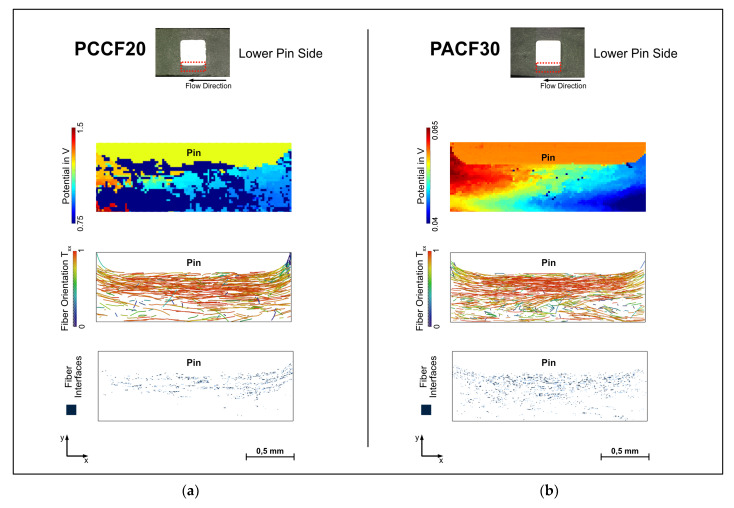
Comparison of the measured potential fields and the fiber orientation at the lower pin sides of the specimen PC CF20_4 mm (**a**) and PA CF30_5 mm (**b**).

On the lower pin side (Figure 19), there are many large areas in the 20 wt.% specimen not connected to the conductive network. This can be clearly deduced from the fiber orientation. In contrast, in the 30 wt.% specimen, there are practically no areas where the conductive network of the carbon fibers is interrupted. A higher filling degree enables a good cross-linking of the carbon fibers throughout the whole specimen, at least at the surface.

What is almost the same for both filling levels is the poor bonding to the pin, since the carbon fibers lie parallel to the pin, similar to the near gate side.

**Figure 20:**
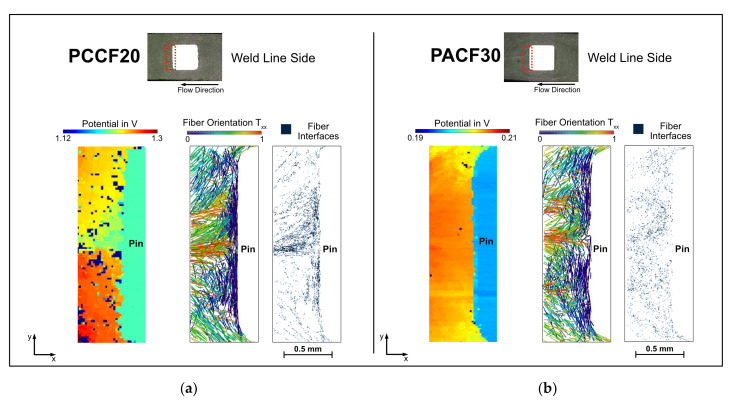
Comparison of the measured potential fields and the fiber orientation at the weld line sides of the specimen PC CF20_4 mm (**a**) and PA CF30_5 mm (**b**).

At the weld line side (Figure 20), however, the carbon fibers are well bonded to the pin for the PC CF20 specimen (indicated by the small potential difference between pin and weld line side). The bonding of the weld line and pin of the PA CF30 specimen is poor, likely due to the observed gap formation (Figure 16). Although the specific conductivity of a higher filling degree should be beneficial also for the contact resistance, the combination with polyamide seems unfavorable. Regarding the PC CF20 specimen, it can be clearly seen that the carbon fibers are perpendicular to the pin, especially in the area where the melt meets and forms the weld line. Here, the potential difference is almost zero. This result is interesting because the weld line area actually provides a worse resistivity compared to the near gate area, but for contacting a metal pin, the weld line side is clearly preferable.

## 4. Conclusions

In this study, carbon fiber reinforced plastics (CFRP) with polyamide 6 and polycarbonate matrix were investigated by means of electrically conductive assembly injection molded specimens. The main aspects here involved the structural properties as a function of the phenomena induced by the injection molding process with the resulting anisotropic electrical properties and, especially, the underlying measurement method. Based on all results and their corresponding interpretations, the following conclusions can be drawn:

- By designing the cavity of the injection mold and purposeful placement of the contact pins, it was possible to achieve specific microstructural properties of the CFRP compound samples, particularly with regard to the fiber orientation. The focus was on the areas close to the contact on the different end faces, as well as the plastic compound volume before and after flowing around the insert.
- These fiber structures could be detected and quantitatively visualized by classical methods such as reflected light microscopy and also X-ray microtomography.
- For the electrical characterization, a suitable examination area was identified, exposed and prepared for the investigations.
- In order to investigate and electrically distinguish the areas near and far from the gate, the total and partial resistances of the specimens were measured.
- For a more accurate non-destructive electrical characterization of the examination area, a potential field measurement method based on scanning the specimen surface was applied. The focus was on the investigation of the local anisotropic electrical properties and their connection with the structural properties.
- As a result of the resistance measurements, it was determined that the near gate area of the specimen has a lower resistance than the weld line area. This is consistent with the observation of the fibers, because they are clearly more consistently oriented in the near gate area than in the weld line area.
- The weld line area is largely defined by the shape of the weld line itself and the free jet formation, which results in a more chaotic fiber orientation and distribution.
- The near gate area can be divided into different layers. Within these layers, the fiber orientation is relatively uniform and ordered, but the layers differ from each other and therefore have different current densities.
- The differences in the ordered structure of the fibers in the near gate area and the disordered arrangement in the weld line area are also evident in the equipotential lines.
- When looking at the pin area, it becomes clear that there is a relatively large shrinkage of the composite material at the contact surface in the PA CF30 specimen. This poor bonding of the contact pin to the plastic is also evident from the high partial resistances measured compared to the total resistance.
- The pin area can be divided into three subareas (pin sides), which differ in terms of their fiber orientation in relation to the contact surface of the pin.
- The three sides of the contact pin area could be successfully investigated with respect to their contacting and the measured potential fields could be correlated with the fiber orientations.
- The isolated spots in the plastic matrix, which are not connected to the conductive network, can be detected by the potential field measurement method. When comparing PC CF20 and PA CF30, clear differences due to the different filler concentrations can be seen with regard to the number of isolated spots.
- Although the weld line area has a poorer specific conductivity, the weld line side is more suitable when it comes to good electrical contact with metal pins.
- The measurement methods can be optimized to obtain even more detailed results. For this purpose, current methodological difficulties regarding the potential field method should be taken into account with respect to the measurability of materials with a low degree of filling. Furthermore, the evaluation of the tomographic data can be optimized and extended in order to obtain even more representative data. For the theoretical procedure in the case presented here, it is essential to solve Ohm’s law, for which an approximation of the location-dependent specific electrical conductivity is provided.

In the future, it will be possible to build on the obtained results and further research the contacting between CFRPs and metal pins. The correlation between the potential field measurement and the fiber orientation and distribution obtained from X-ray tomography allows, among other things, further conclusions to be drawn about the contact situation and the anisotropic conductivity distribution.

## Figures and Tables

**Table 1:** Dimensions of feather keys used as electrical contact pins according to DIN 6885.

Width *w*	Nominal dimension Mean dimension ^1^	in mm	2 1.985 ± 4.9 × 10^3^
Height *h*	Nominal dimension Mean dimension ^1^	in mm	2 1.985 ± 4.9 × 10^3^

^1^ Measured dimensions and corresponding standard deviation from a sample of ten.

**Table 2:** Sputtering parameters of the contact pin coating process.

Sputtering target		Gold
Operating gas		Argon
Operating distance	in mm	50
Operating pressure	in mbar	0.05
Sputtering current	in mA	30
Sputtering time	in s	40
Layer thickness (approx.)	in nm	10

**Table 3:** Injection molding parameters.

		PC CF20	PA CF30
Cylinder temperature	in °C	290	260
Mold temperature	in °C	100	87
Volume flow rate	in cm^3^/s	24	24
Injection time	in s	0.24	0.24
Packing pressure point 1	in bar	600	400
Packing pressure point 2	in bar	400	380
Packing time	in s	11	11
Cooling time	in s	30	30

**Table 4:** XFiber extension settings (Avizo 9.4).

		PC CF20	PA CF30
Cylinder length	in μm	38	38
Angular sampling		5	5
Mask cylinder radius	in μm	7	7
Outer cylinder radius	in μm	3	3
Minimum seed correlation		205	195
Minimum continuation quality		112	122
Direction coefficient		0.3	0.3
Minimum distance	in μm	2	2
Minimum length	in μm	38	38

**Table 5:** Positioning accuracies of the *X*-, *Y*- and *Z*-axes.

*X*-Axis	*Y*-Axis	*Z*-Axis
<±20 µm	<±20 µm	<±10 µm

**Table 6:** Electrical resistances.

Electrical Resistance	PC CF20_4 mm	PC CF20_5 mm	PA CF30_5 mm
*R_total_* in Ω	163	119	3.9
*R*_1_ in Ω	134	91	7.7
*R*_2_ in Ω	84	67	7.3

## Data Availability

Not applicable.
